# A Flexible Approach for Human Activity Recognition Using Artificial Hydrocarbon Networks

**DOI:** 10.3390/s16111715

**Published:** 2016-10-25

**Authors:** Hiram Ponce, Luis Miralles-Pechuán, María de Lourdes Martínez-Villaseñor

**Affiliations:** Faculty of Engineering, Universidad Panamericana, 03920 Mexico City, Mexico; lmiralles@up.edu.mx (L.M.-P.); lmartine@up.edu.mx (M.d.L.M.-V.)

**Keywords:** artificial organic networks, artificial hydrocarbon networks, flexible human activity recognition, supervised machine learning, wearable sensors, flexibility

## Abstract

Physical activity recognition based on sensors is a growing area of interest given the great advances in wearable sensors. Applications in various domains are taking advantage of the ease of obtaining data to monitor personal activities and behavior in order to deliver proactive and personalized services. Although many activity recognition systems have been developed for more than two decades, there are still open issues to be tackled with new techniques. We address in this paper one of the main challenges of human activity recognition: Flexibility. Our goal in this work is to present artificial hydrocarbon networks as a novel flexible approach in a human activity recognition system. In order to evaluate the performance of artificial hydrocarbon networks based classifier, experimentation was designed for user-independent, and also for user-dependent case scenarios. Our results demonstrate that artificial hydrocarbon networks classifier is flexible enough to be used when building a human activity recognition system with either user-dependent or user-independent approaches.

## 1. Introduction

Physical activity recognition based on sensors is a growing area of interest given the great advances in wearable sensors, and the common use of smartphone with powerful embedded sensors. Wearable sensors are getting less obtrusive allowing using sensors for longer periods of time. Applications in various domains are taking advantage of the ease of obtaining data to monitor personal activities and behavior in order to deliver proactive and personalized services.

Although many activity recognition systems have been developed for more than two decades, there are still open issues to be tackled with new techniques. Lara et al. [1] envisions six designing challenges for activity recognition: (1) the selection of attributes and sensors; (2) the construction of portable, unobtrusive, and inexpensive data acquisition system; (3) the design of feature extraction and inference methods; (4) data collection under realistic conditions; (5) the flexibility to support new users without the need to re-train the system; and (6) energy consumption. This list of challenges is not exhaustive given that there are other challenges common to various activity recognition scenarios. Recognizing concurrent activities, recognizing interleaved activities, ambiguity of interpretation, and multiple residents are challenges of the nature of human activities defined by [2] needed to be addressed also. We address in this paper one of the main challenges of human activity recognition (HAR) mentioned above: Flexibility. We adopted the flexibility in HAR defined by Lara et al. [1]. In [1], flexibility is contemplated as the ability of the classifier to support new users without the need to collect additional data of the user and re-train the system.

Flexibility in activity recognition classifiers can be considered regarding different aspects. For example, flexibility is considered by [3] as the ability of the classifier to recognize different kinds of activities: Common daily activities, activities specific for a certain group of persons, or activities that are rarely performed. Bulling et al. [4] is more interested in generalization ability of the activity recognition classifier. They categorized activity recognition system taking into account if the level of generalization is user independent, user specific and robust to cope with temporal variations. In summary, classifiers must be able to cope with multiple persons performing activities on multiple days, and in multiple runs containing repetitions of the set of activities.

Human activity recognition systems generate generic or universal models using training data from several users. These models are then applied to new users without the need to retrain the generic model. Other systems are more focused in specific users that generate personal models that perform the train-test processes only with data of the subject of interest. Recently, personalization of physical activity recognition approaches deal with a new subject from whom data is not available in training phase in a subject- independent system [5]. Nevertheless, personalization approaches only address one aspect of generalization.

In previous work, we presented the results of the first tests applying artificial hydrocarbon networks (AHN) for human activity recognition task in [6], using raw sensor data of a public dataset containing five basic and distinctive activity classes (sitting-down, standing-up, standing, walking, and sitting). We compared models generated with ten well-known supervised learning methods against AHN method focusing in the comparison against deep learning method. From this preliminary analysis we concluded that AHN are suitable for activity recognition.

Results of a thorough experimental analysis to prove that AHN classifier are very competitive and robust for physical activity recognition was presented in [7]. In that paper we focused in one challenge of HAR: To deal with incomplete noise tolerance. Four experiments were designed using raw data and one window-based approach of another public database with 18 more complex activity classes.

Our goal in this work is to present artificial hydrocarbon networks as a flexible approach in a human activity recognition system. We consider flexibility of the approach mainly regarding the ability to support new users (user-independent). We are also concerned the ability to support variations of the same subject in a user-specific approach, and the ability to handle new or irrelevant activities, so we designed some experiments regarding these issues. Since we are using a public dataset for experimentation, real-time variations due to sensors or user behavior are out of the scope of this work.

In order to evaluate the performance of artificial hydrocarbon networks based classifier, three kinds of experiments were designed using Attal et al.’s methodology [8]. For each case scenario, the performance of the proposed artificial hydrocarbon networks based classifier was compared with eighteen supervised techniques frequently used in activity recognition systems. Case 1 experiment was designed to assess the performance for all individuals, case 2 experiment assesses the performance of our classifier for user-independent scenario. The first case experiment used cross validation evaluation schema, and the second case used leave-one-subject-out validation. The third experiment (case 3) was designed to test the performance of our classifier for user-dependent scenario. In this case, the classifiers were trained and tested for each individual with her/his own data, and average accuracy and standard deviation was computed.

The rest of the paper is as follows. Section 2 discusses related work in flexibility in human activity recognition systems. A brief description of artificial hydrocarbon networks (AHN) technique is presented in Section 3. Our proposed AHN-classifier is presented in Section 4. In Section 5, experimentation is presented, and in Section 6, the results are discussed. Conclusions and directions for future research are described in Section 7.

## 2. Flexibility in Human Activity Recognition

Every person performs activities in different manner depending on their characteristics such as age, gender, weight, and health condition. Even the same person can change the way of performing an activity depending in the time of the day, emotional and physical state among other thinks. Therefore, the flexibility of a classifier to cope with this diversity of manners of performing the same activity is still one of the main issues of human activity recognition (HAR) [1]. The measures of wearable sensors gathered from a male elderly, a child or a handicapped person doing the same activity present significant differences.

One of the main characteristics considered when evaluating human activity recognition systems is flexibility [1]. Although flexibility in a HAR classifier is usually thought regarding the generalization ability in recognizing activities for a new person, it is also considered as the ability of recognizing new activities for one person, or even new runs or sessions to prove robustness over time [4]. In other applications, for example in the video surveillance domain [9] it is more important to consider the classifiers flexibility regarding the ability for adding new activities, namely new and unusual events. Bulling et al. [4] include the characteristic generalization in a HAR system. They identify user-independent systems, user –specific systems, and temporal systems. Lara et al. [1] define the classifier flexibility level as user-specific and monolithic (for user-independent).

In the user-specific approach, the system is designed to work with a certain user and to self-adapt to his/her characteristics. Specific approaches are mainly recommended when the users are elderly people, patients with some health problems or disabled. User-dependent systems are frequently used in assisted-living domain given that elderly and people with health problems present differences in their main characteristics that hamper the performance of a generic classifier [8,10,11]. Recently, Capela et al. [12] compared activity recognition with able-bodies and stroke participants, proving that their classifiers performed worse for stroke participants. Regarding their performance, as expected, user-specific models have better performance, but are not generalizable. The main drawback of this approach is that a new model must be done for each user; the system must be retrained.

Unlike the specific approach, user-independent systems need to be flexible enough to work with different users [1]. It is important for this kind of systems to be able to keep good performance if new users arrive. Generic or universal models are created from time series dataset of measured attributes from a small or large set of individuals performing each different activity. Depending on case use scenarios, new users may arrive to the activity recognition process. Too many or new activities can also be performed by individuals, making it difficult for one model to cope with all those differences. One way to solve this problem is to create groups with similar characteristics and/or similar activities performed.

Some systems like [13], carry out subject-dependent and subject-independent analysis to prove that their classification technique is able to cope with multiple persons, but is also well fitted to build a specific oriented model.

Recently, personalization of physical activity recognition has gained interest. Personalization approaches try to deal with the fact that training for activity recognition is usually done on a large number of subjects, and then applied with a new subject from whom data is not available in training phase [5]. Each person has different characteristics that ultimately cause high variance in the activity recognition performance for each subject. Personalization approaches try to cope with these differences adapting the model created with large number of subjects for its application with new users [3,5,14]. In [14], the authors create a model for basic activity recognition based on a decision tree technique, and they change the thresholds of the decision nodes afterwards based on labeled data of each new user. Berchtold et al. [3] present a modular classifier approach based on Recurrent Fuzzy Inference Systems (RFIS). In the later approach the best classifier module is selected from a set of classifiers, and it is adapted to work with new users. In works [3,14], parameters are changed of a general model in order to adapt this universal model to new users. The drawback of this approach is that the general model is either to simple to cope with challenging activity tasks and variety of users, or the model is to complex and therefore entails great computational costs. Reiss [5] presents a different method of personalization in which the general model consists in a set of classifiers weighted the same. A strategy based on weighted majority voting is applied to increase the performance of the model for new users. Instead of retaining classifiers, the method retains only weights reducing the computational complexity.

Personalization of physical activity recognition applications is a valid approach to deal with new subject from whom data is not available in training phase in a subject-independent system. Nevertheless, personalization approaches only address one aspect of generalization [15].

A number of researches have explored transfer learning for activity recognition [16]. Transfer learning is the ability to extend what has been learned in one context to new context [17,18]. This approach allows reusing the knowledge previously obtained in a source to a new target population. Roggen et al. [19] defined a run-time adaptive activity recognition chain (adARC) to deal with variations due to placement of sensors, behavior of the user over time, and sensing infrastructure. This architecture allows adaptation according to the recognition of new conditions of the system. The smartphone-based framework of self-learning schema presented in Guo et al. [20] is able to recognize unpredictable activities without any knowledge in the training dataset. They also support variations in smartphone orientation. Li et al. [21] proposed a generic framework for human motion recognition based on smartphones. They presented features to deal with variations due to sensor position and orientation, and user motion patterns.

Regarding experimentation design, feature selection can help or hinder the flexibility performance of a HAR classifier. Given the great variability in the performance of activities between different subjects, and even in the same subject at different time, features derived from wearable sensors can lead to great variability. “A good feature set should show little variation between repetitions of the same movements and across different subjects but should vary considerably between different activities” [22]. It is very important to find the best subset of features that combined deliver the best predictors.

Regarding the classifier evaluation scheme, subject-dependent and subject-independent methods of evaluation analysis have been used [13].

Preece et al. [22] commented that cross-validation can be done in evaluations between different subjects and within-subject. In user–independent (or between-subject) oriented systems, training is made with almost every subject and test with leave one or a few subjects out. The train-test process is repeated until all subjects have been tested. For the within-subject case, train-test process is made only with the data of a subject, and this process is repeated for data of all subjects available. Average accuracy must be calculated from the results of train-test repetitions in both cases. Lara et al. [1] describe similar evaluation schemes in order to assess the flexibility power of a classifier for each kind of generalization. They state that cross validation or leave-one-out validation schemes are used in user-independent analysis. “Leave-one-person-out is used to assess generalization to an unseen user for a user-independent recognition system” [5].

## 3. Artificial Hydrocarbon Networks

Artificial hydrocarbon networks (AHN) is a supervised learning method inspired in organic chemistry in order to simulate the chemical rules involved within organic molecules, representing the structure and behavior of data [23,24].

Currently, this method inherits from a general framework of learning algorithms so-called artificial organic networks that proposes two representations of artificial organic molecules: A graph structure related to their physical properties, and a mathematical model behavior related to their chemical properties. The main characteristic of artificial organic networks is packaging information in modules called molecules. These packages are then organized and optimized using heuristic mechanisms based on chemical energy. For readability, Table 1 summarizes the description of chemical-based terms of the artificial organic networks framework and their meanings in the computational AHN technique described below [23].

To this end, artificial organic networks, as well as artificial hydrocarbon networks, allow [23,25]: Modularity and organization of information, inheritance of packaging information, and structural stability of data packages. A detailed description of the artificial organic networks framework can be found in [23].

### 3.1. Description of the AHN-Algorithm

**Figure 1:**
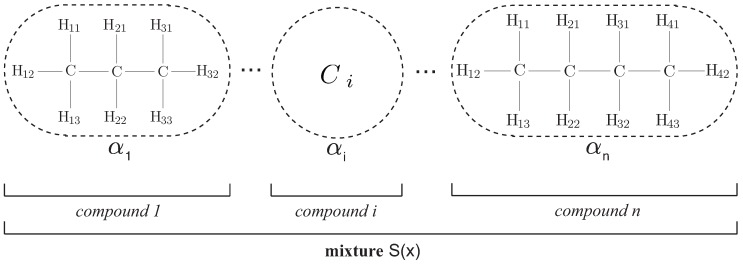
Structure of an artificial hydrocarbon network using saturated and linear chains of molecules [26]. Throughout this work, the topology of the proposed classifier considers one hydrocarbon compound. Reprinted from Publication Expert Systems with Applications, 42 (22), Hiram Ponce, Pedro Ponce, Héctor Bastida, Arturo Molina, A novel robust liquid level controller for coupled tanks systems using artificial hydrocarbon networks, 8858–8867, Copyright (2015), with permission from Elsevier.

Artificial hydrocarbon networks algorithm (see Figure 1) is inspired in chemical hydrocarbon compounds; thus, this algorithm is only composed of hydrogen and carbon elements that can be linked together with at most one and four atoms, respectively. In this algorithm, linking them in a specific way forms molecules which they are primitive units of information so-called CH-molecules [23]. In fact, these molecules define a mathematical function *φ* representing the behavior of the CH-molecule, or $CH_{k}$, as expressed in (1); where, $\sigma_{r}\in R$ is called the carbon value, $H_{i}\in C$ is the *i*-th hydrogen atom attached to the carbon atom, *k* represents the number of hydrogen atoms in the CH-molecule, and $x=(x_{1},\ldots ,x_{p})$ is the input vector with *p* features.

(1) $$\varphi \left(x\right)=\sum_{r=1}^{p}\sigma_{r}\prod_{i=1}^{k\leq 4}\left(x-H_{i}\right)$$

Two or more unsaturated molecules, i.e., k<4, can be joined together in order to form artificial hydrocarbon compounds. Different compounds have been defined in literature [23], and the simplest of those is the saturated and linear chain of molecules like in (2); where, the line symbol represents a simple bond between two molecules. In fact, if there are *n* CH-molecules, then the compound will have two $CH_{3}$ and (n−2) $CH_{2}$ molecules [25,26]. Then, a function ψ∈R is associated to the behavior of the artificial hydrocarbon compound, e.g., the piecewise function [23,27], as expressed in (3); where, $L_{t}$ represents the *t*-th bound that limits the action of a CH-molecule over the input space by transforming the bounds into centers $M_{c,j}$. In that sense, if the input domain is in the interval $x\in [L_{min},L_{max}]$, then $L_{0}=L_{min}$ and $L_{n}=L_{max}$, and the *j*-th CH-molecule is centered at $M_{c,j}=\left(L_{j-1}+L_{j}\right)/2$, for all j=1,…,n [23].

(2) $$CH_{3}-CH_{2}-\cdots -CH_{2}-CH_{3}$$

(3) $$\psi \left(x\right)=\left\{\begin{array}{cc} \varphi_{1}\left(x\right) & 1=\arg\min_{t}\left(x-M_{c,t}\right)\\ \cdots & \cdots \\ \varphi_{n}\left(x\right) & n=\arg\min_{t}\left(x-M_{c,t}\right) \end{array}\right.$$

In addition, bounds are computed using the distance $r_{j}$, as (4), between two adjacent molecules, i.e., $r_{j}=∥L_{j}-L_{j-1}∥$ with j=1,…,n. A gradient descent method based on the energy of the adjacent molecules ($E_{j-1}$ and $E_{j}$) is used to calculate the distances as in (5); where, 0<η<1 is the learning rate parameter [23,25]. For implementability, the energy of molecules is computed using a loss function [23,25]. In this work, the least squares estimates (LSE) was used to compute the energy of molecules.

(4) $$r_{j}=r_{j}+\Delta r_{j}$$

(5) $$\Delta r_{j}=-\eta \left(E_{j-1}-E_{j}\right)$$

Several artificial hydrocarbon compounds can interact among them in definite ratios, so-called stoichiometric coefficients, forming a mixture S(x)∈R. To this end, a mixture is represented as shown in (6); where, *c* represents the number of compounds in the mixture and $\alpha_{i}\in R$ is a set of stoichiometric coefficients [23].

(6) $$S\left(x\right)=\sum_{i=1}^{c}\alpha_{i}\psi_{i}\left(x\right)$$

Formally, an artificial hydrocarbon network is a mixture of artificial hydrocarbon compounds (see Figure 1) each one computed using a chemical-based heuristic rule, expressed in the so-called AHN-algorithm [23,25]. Throughout this work, an artificial hydrocarbon network considers one compound, such that c=1 and $S\left(x\right)=\psi_{1}\left(x\right)$. As noted, the AHN-algorithm is reduced to Algorithm 1 that uses saturated and linear hydrocarbon compounds.

At first, the AHN-algorithm initializes an empty compound AHN={}. Then, a new compound *C* with *n* CH-molecules is created as well as a set of random distances $r_{j}$. While the difference between real and estimated values are greater than a tolerance value ϵ>0, the data set is partitioned into *n* subsets $\Sigma_{j}$ using the set of bounds $L_{0},L_{j}$ generated with the intermolecular distances. With each subset, the hydrogen and carbon values of the molecular behavior are computed using the LSE method. Then, the compound behavior is assembled and the distances $r_{j}$ are updated using the error values computed in the LSE method. When the difference between real and estimated values fulfills the tolerance value, the AHN compound is updated with *C* and its behavior *ψ* such that AHN=〈C,ψ〉. A detailed description of the AHN-algorithm can be found in [23,25]. Also, Appendix A shows a numerical example of training and testing artificial hydrocarbon networks.
**Algorithm 1** AHN-Algorithm for saturated and linear hydrocarbon compounds, adapted from [23].**Input:** the training data set Σ=(x,y), the number of molecules in the compound n≥2, the learning rate *η* and the tolerance value ϵ>0.**Output:** the trained compound AHN. Initialize an empty compound AHN={}.Create a new compound *C* of *n* CH-molecules like: $CH_{3}-\underset{n-2}{\underset{︸}{CH_{2}-\cdots -CH_{2}}}-CH_{3}$. Randomly initialize the set of distances $r_{j}$ for j=1,…,n.**while**
∥y−ψ∥>ϵ
**do** Determine all bounds $L_{j}$ using $r_{j}$ by using $L_{j}=L_{j-1}+r_{j}$ with $L_{0}=L_{min}$, $L_{n}=L_{max}$ and all $L_{j}\leq L_{max}$. Split Σ in *n* subsets using bounds $L_{j}$, i.e., $\Sigma_{t}=\left(x^{\left(q\right)},y^{\left(q\right)}\right)$ such that $t=\arg\min_{j}(∥x^{\left(q\right)}-\frac{L_{j-1}+L_{j}}{2}∥)$ and j=1,⋯,n. **for each** molecule *j* in *C*
**do**  Compute all parameters $H_{i}$ and $\sigma_{r}$ of function $\varphi_{j}\left(x\right)=\sum_{r=1}^{p}\sigma_{r}\prod_{i=1}^{k\leq 4}\left(x-H_{i}\right)$ using the LSE method and the partition $\Sigma_{j}$.  Store the error value $E_{j}$ when calculating the LSE metric. **end-for** Build the compound behavior $\psi \left(x\right)=\left\{\begin{array}{cc} \varphi_{1}\left(x\right) & 1=\arg\min_{t}\left(x-M_{c,t}\right)\\ \cdots & \cdots \\ \varphi_{n}\left(x\right) & n=\arg\min_{t}\left(x-M_{c,t}\right) \end{array}\right.$. Compute all $\Delta r_{j}=-\eta \left(E_{j-1}-E_{j}\right)$ with $E_{0}=0$. Update all distances $r_{j}=r_{j}+\Delta r_{j}$.**end-while**Update AHN with *C* and *ψ*.**return**
AHN

### 3.2. Properties of Artificial Hydrocarbon Networks

The artificial hydrocarbon networks algorithm is characterized by several properties that are very useful when considering regression and classification problems, such as [7,23,26]: Stability, robustness, packaging data and parameter interpretability. Particularly, stability implies that the AHN-algorithm minimizes the changes in its output response when inputs change slightly [7,23], promoting the usage of the artificial hydrocarbon networks as a supervised learning method. In addition, robustness considers that the AHN-algorithm can deal with uncertain and noisy data which implies that it behaves as a filtering information system. For example, it has been used in audio filtering [23,27], and ensembles of artificial hydrocarbon networks with fuzzy inference systems have been successfully employed as intelligent control systems [24,26]. Packaging data is another property of the AHN-algorithm. In fact, this characteristic enables to compute molecular structures into the algorithm in the sense that similar data with similar capabilities are clustered together [23]. In fact, this property intuitively reveals that data is not only packaged by its features, but also by its tendency. Lastly, parameter interpretability refers to that bounds, intermolecular distances and hydrogen values can be useful as metadata to partially understand underlying information or to extract features. For example, the AHN-algorithm has been used in facial recognition approaches when using its parameters as metadata information [23].

Furthermore, the artificial hydrocarbon networks algorithm can be contrasted with other learning models. For instance, it is a supervised, parametric, nondeterministic and multivariate learning algorithm. It means that backpropagation-based multilayer artificial neural networks and support vector machines are close related to artificial hydrocarbon networks in terms of supervised learning and non-probabilistic models used for regression and classification problems. In fact, in [23] authors analyze the location of the AHN-algorithm in the space of learning models, concluding that it is located between regression algorithms, e.g., linear regression and general regression based-learners, and clustering algorithms like *k*-nearest neighbors, *k*-means algorithm and fuzzy clustering means. Also, like-smoothers models are not far away from the AHN-algorithm, supporting the robustness property of the latter. To this end, random forest and decision trees models are probabilistic algorithms differing from the artificial hydrocarbon networks algorithm. A detailed comparison of the AHN-algorithm with other learning models can be seen in [23].

## 4. Description of the Artificial Hydrocarbon Networks Based Classifier

**Figure 2:**
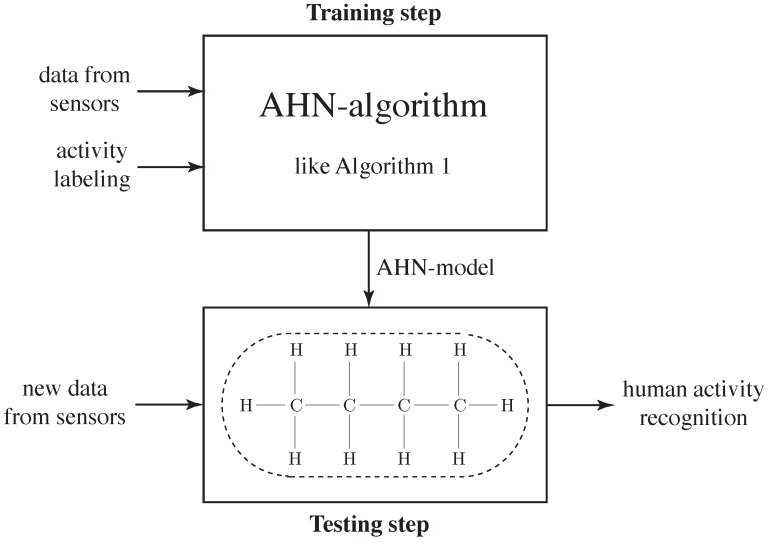
Diagram of the proposed artificial hydrocarbon network based classifier (AHN-classifier). First, reduced feature set is used to train the AHN-model, then it is used as AHN-classifier in the testing step.

This work considers training and using an AHN-classifier as a flexible approach in human activity recognition systems. In fact, this AHN-classifier is computed and employed in two steps: Training-and-testing and implementation, as shown in Figure 2. Previous work in this direction can be found in [6,7].

Currently, the AHN-classifier considers that sensor data has already processed in *N* features $x_{i}$ for all i=1,…,N, and has organized in *Q* samples, each one associated to its proper label $y_{j}$ representing the *j*th activity in the set of all possible activities *Y* for j=1,…,J; where, *J* is the number of different activities in the data set. Thus, samples are composed of features and labels as (N+1)-tuples of the form ${\left(x_{1},\ldots ,x_{N},y_{j}\right)}^{q}$ for all q=1,…,Q.

Considering that there is a dataset of *Q* samples of the form defined above, then the AHN-classifier is built and trained using the AHN-algorithm shown in Algorithm 1. It should be noted that this proposal is using a simplified version of artificial hydrocarbon networks. Thus, the AHN-classifier is composed of one saturated and linear hydrocarbon compound, i.e., no mixtures were considered (see Figure 1 for a hydrocarbon compound reference). In that sense, the inputs of the AHN-algorithm are the following: The training dataset Σ is a subset of *R* samples, from the original dataset, as (7), the number of molecules *n* in the hydrocarbon compound is proposed to be the number of different activities (n=J), and the learning rate 0<η<1 and the tolerance value *ϵ* are positive numbers selected manually. Notice that the number of molecules in the compound is an empirical value, thus no pairing between classes and molecules occurs. At last, the AHN-algorithm will compute all parameters in the AHN-classifier: Hydrogen and carbon values, as well as the bounds of molecules.

(7) $$\Sigma =\left[\begin{array}{c} {\left(x_{1},\ldots ,x_{N},y_{j}\right)}^{1}\\ \vdots \\ {\left(x_{1},\ldots ,x_{N},y_{j}\right)}^{R} \end{array}\right]$$

For testing and validating the AHN-classifier, the remaining samples *P* from the original data set (i.e., such that Q=P+R) conforms the testing data set. Then, the testing data set is introduced to the AHN-classifier, previously computed, and the output response is rounded in order to obtain whole numbers as labels. If output values were out the permitted labels, they were considered as the nearest defining label. Lastly, validation of the classifier is calculated using some metrics. Moreover, new sample data can be also used in the AHN-classifier for recognizing and monitoring a human activity based on the corresponding features.

## 5. Experimentation

**Figure 3:**
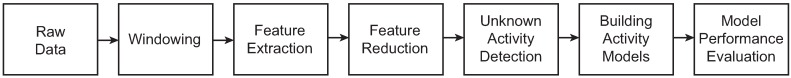
Methodology implemented in the case study for HAR systems.

A case study of human activity recognition was implemented using a public dataset in order to measure how well the proposed AHN-classifier performs as a flexible approach in HAR systems. We adopted the activity recognition chain (ARC) approach described by Bulling et al. [4] and we also added an unknown-activity detection module in order to discriminate possible new or irrelevant activities that might lead in misclassification. Our approach performs the following stages: (i) data acquisition; (ii) signal preprocessing and segmentation, e.g., windowing; (iii) feature extraction; (iv) feature reduction; (v) building an unknown-activity detector; (vi) building activity models; and (vii) classification or activity evaluation. Figure 3 shows the methodology of the HAR system of this case study.

### 5.1. Dataset Description

**Figure 4:**
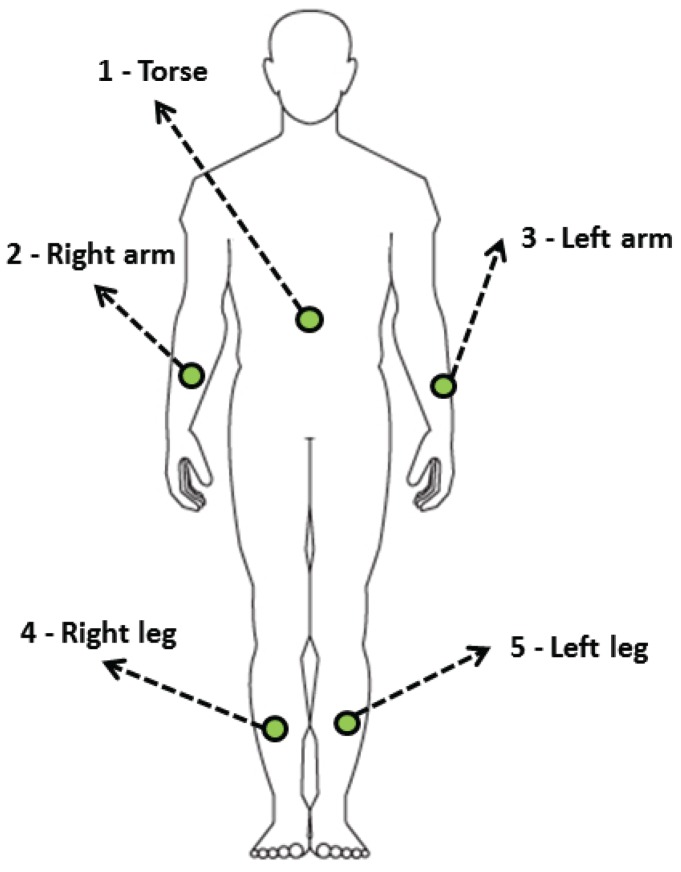
Location of the five wearable IMUs used in the dataset.

This case study employs a dataset provided by the Bilkent University from Ankara, Turkey [28]. It consists on a set of 45 raw signals from five inertial measurement units (IMUs) placed in the body of eight different subjects, performing nineteen different activities. In fact, each IMU is composed of three 3-axes sensors: An accelerometer, a gyroscope, and a magnetometer. In addition, Figure 4 shows the position of the IMUs: One at the torse, two at the arms and two at the legs.

The nineteen activities carried out by the subjects are [28]: (1) sitting; (2) standing; (3) lying on back; (4) lying on right side; (5) ascending stairs; (6) descending stairs; (7) standing in an elevator still; (8) moving around in an elevator; (9) walking in a parking lot; (10) walking on a treadmill with a speed of 4 km/h in flat; (11) walking on a treadmill with a speed of 4 km/h and 15 degree inclined positions; (12) running on a treadmill with a speed of 8 km/h; (13) exercising on a stepper; (14) exercising on a cross trainer; (15) cycling on an exercise bike in horizontal positions; (16) cycling on an exercise bike in vertical positions; (17) rowing; (18) jumping; and (19) playing basketball.

We used the public dataset [28] given that each activity was performed by the subjects in their own style. This allows inter-subject variability. It is also correctly labeled and segmented by subject and by activity. These segmentations permit to easily design different experimental datasets. The limitation of this dataset is that it does not include intra-subject variability.

### 5.2. Windowing and Feature Extraction

We apply a windowing approach to the entire dataset of raw signals. In particular, we select windows of 5 s in size without overlapping. Then, we extract 18 features for each channel based on literature: 12 features in time domain as shown in Table 2, and 6 features in frequency domain as shown in Table 3. Currently, each window is composed of 125 raw samples, and there are 1140 windows per subject. Considering that each activity is performed during 5 min by each subject, then there are 60 windows per activity.

### 5.3. Feature Reduction

Considering that there are 45 channels of raw signals and 18 features per channel, then the total number of features extracted is 810. Due to the fact that the latter demands high computational resources, a feature reduction procedure was applied using the well-known principal components analysis (PCA) [35].

**Figure 5:**
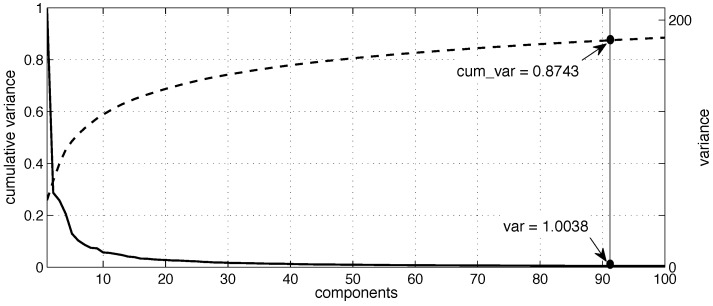
A subset of the first one-hundred components calculated by the PCA method: Variance values shown in straight line, and cumulative variance shown in dashed line.

Currently, PCA transforms a high dimensional domain into a lower dimensional domain by applying a linear combination of weighted features. In that sense, we applied PCA to the feature set and we obtain a reduced feature set of so-called components [35]. In order to select the optimal number of components, we chose the eigenvalue criterion or the Kaiser criterion [36], one of the most commonly used criteria for solving the number of components problem in PCA that consists of retaining any component with a variance value greater than 1. Thus, the components were sorted in descending order, finding that the first 91 components have variance value greater than one (representing the 87.43% of the feature set), as shown in Figure 5. To this end, the reduced feature set of the first 91 components was employed in this case study to build the activity models, as described below.

### 5.4. Unknown-Activity Detection Module

We developed a module to detect new and/or irrelevant activities inspired in the methodology of Guo et al. [20]. This module performs a rough classification of reduced feature vectors in *known* and *unknown* activities using an AHN-based classifier. If an instance is considered *unknown*, it will be stored for future manual tagging. Otherwise, the instance is processed normally. It should be noted that this module is a first and independent classifier that roughly determines if a reduced feature vector would be an already known activity in order to let it continue in the workflow.

In order to validate this module, we selected five different activities (sitting, lying on back, ascending stairs, walking in a parking lot and exercising on a stepper) coming from all the subjects in the dataset avoiding user-specific training. Then, we used 70% of them to build the AHN-classifier. From (3), it can be seen that each molecule has an associated parameter referring to its center $M_{c,j}$. Then, these centers $M_{c,j}$ can be used as the centers, namely $v_{j}$ for all j=1,⋯,5, of these clusters/activities. Then, we measured the distance of each training sample to the nearest center, and we computed the mean *m* and standard deviation *σ* of these distances. After that, the unknown activity detection module was developed using the heuristic h(x) as expressed in (8); where, *x* is the input (i.e., the reduced feature vector representing the testing sample), *d* is the $L_{2}$-norm distance, and $v_{j}$ is the *j*-th center computed before when training the AHN-classifier. In a nutshell, h(x) determines if the input *x* is near at least to one of the clusters defined by the training activities (h(x)=1) and then is a *known* activity. If not (h(x)=0), then the input *x* is an *unknown* activity.

(8) $$h\left(x\right)=\left\{\begin{array}{cc} 1 & \min\{d\left(x,v_{c}\right)\}\leq m+1.5\sigma \\ 0 & \mathrm{otherwise} \end{array}\right.$$

Four unknown activities were selected as part of the testing set (i.e., cycling on an exercise bike in horizontal positions, jumping, walking on a treadmill with a speed of 4 km/h in flat and lying on right side) as well as the remaining 30% of the known activities. Table 4 shows the accuracy of this module for detecting *known* and *unknown* activities. In terms of the *known* activities, this module recognizes it with a mean accuracy of 87.4%. However, cycling on an exercise bike and walking on a treadmill with a speed of 4 km/h in flat activities were misclassified. In the first activity, the AHN-classifier got confused between exercising on a stepper and cycling on an exercise bike; while the latter can be explained since it is very similar to the known activity walking in a parking lot. Lying on right side activity was well classified.

For comparison purposes, we also designed a similar classifier based on the *k*-means method since it calculates centers $v_{j}$ of *known* clusters/activities. Table 4 summarizes its results. It can be seen that the module can classify *known* activities with 92.4% in average. In terms of the *unknown* activities, most of them were classified with 98.6% in average except the activity walking on a treadmill with a speed of 4 km/h in flat. This misclassification can be explained since the latter is very similar to the known activity walking in a parking lot. As noted, both classifiers obtain similar performance accuracy on *known* activities. In terms of *unknown* activities, there is a similar tendency, except on the cycling on an exercise bike activity. Since the activities are well classified and similar *unknown* activities are recognized as the *known*-like activities by the module using AHN or *k*-means, this methodology is proposed to be used before more accurate human activity classifier models.

Finally, this experiment opens the possibility to use the same AHN-classifier for both human activity recognition (using the output response of artificial hydrocarbon networks) and unknown-activity detection (using the parameter interpretability of the center of molecules).

### 5.5. Building Supervised Activity Models

To compare our proposed AHN-classifier, we choose eighteen supervised methods aiming to evaluate the performance of artificial hydrocarbon networks as classifier over HAR systems in both user-independent and user-dependent approaches.

The following supervised learning methods were selected, i.e., supported in reviewed literature [1,22,37,38], to build activity models: stochastic gradient boosting (SGB), AdaBoost (AB), C4.5 decision trees (DT4), C5.0 decision trees (DT5), rule-based classifier (RBC), single rule classification (SRC), support vector machines with basis function kernel (SVM-BF), random forest (RF), *k*-nearest neighbors (KNN), penalized discriminant analysis (PDA), mixture discriminant analysis (MDA), shrinkage discriminant analysis (SDA), multivariate adaptive regression splines (MARS), naive Bayes (NB), multilayer feedforward artificial neural networks (ANN), model averaged artificial neural networks (MA-ANN), nearest shrunken centroids (NSC), and deep learning (DL) using a deep neural networks (DNN) approach. The caret package and other libraries in R were employed to build suitable activity models. Table 5 summarizes the configuration parameters of these models. For reproducibility, we set a seed value, seed=123, when building the models.

In order to build these activity models, three different cases were considered in order to measure and validate the performance of the AHN-classifier in flexibility, as follows:

- *Case 1: All subjects using cross-validation.* This experiment uses 70% of the reduced feature set as the training set and 30% as the testing set, in order to validate how well the AHN-classifier performs for all users. To obtain the best model configuration, we previously used 10-fold cross-validation and 5 repetitions in the training set.
- *Case 2: User-independent performing leave-one subject-out.* This experiment is based on the well-known leave-one subject-out technique [1], aiming to prove how well is the AHN-classifier to predict activities in new subjects. In fact, we build eight models by training each model with information from seven subjects and leaving one subject out. Then, the latter not used in the training step is employed to test the performance of the classifier. Then, the overall performance of classifiers is measured as an average of the eight models.
- *Case 3: User-dependent performing cross-validation within a subject.* This experiment considers building eight different models from each of the subjects in order to measure the performance of the AHN-classifier in a user-specific approach. For each subject, 70% of the feature set is used as the training set and the 30% of it is used as the testing set. Then, the overall performance of classifiers is measured as an average of the eight models.

To this end, the experiments were executed in a computer Intel Core^TM^i5-2400 with CPU at 3.10 GHz and 16 GB-RAM over Windows 7 Pro, Service Pack 1 64-bits operating system.

### 5.6. Metrics

We use different metrics to evaluate the performance of the AHN-classifier in comparison with the other supervised classifiers, such as: accuracy, sensitivity, specificity, precision and $F_{1}\;score$ [39]. Notice that the reduced feature set contains the same size samples of each class, so it is balanced.

Other metrics are computed as well (Table 5): trainingtime specifies the training time (in seconds) to build and train a model, and testingtime specifies the evaluation time of an input sample (in milliseconds).

## 6. Results and Discussion

This section presents the results of the comparison between the proposed AHN-classifier and other eighteen supervised methods as a flexible approach for human activity recognition systems. Then, a discussion is also presented.

### 6.1. Case 1: All Subjects Using Cross-Validation

**Figure 6:**
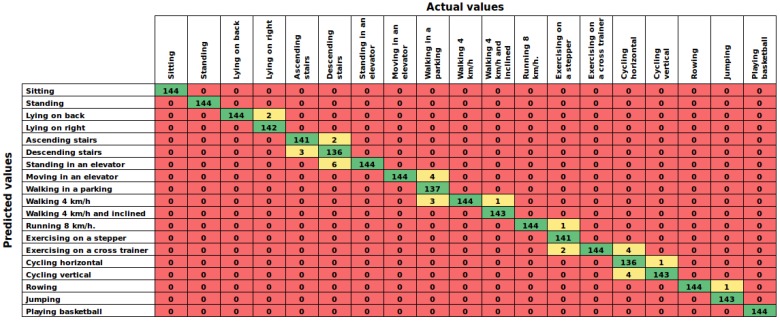
Results of case 1: Confusion matrix of the AHN-classifier in the performance for all subjects using cross-validation. Numbers represent window counts.

A cross-validation with 10-folds and 5-repetitions was computed in the training step to obtain a suitable model. Table 6 summarizes the results of this experiment sorted in descending order by accuracy. Additionally, Figure 6 shows the confusion matrix of the proposed AHN-classifier. As noted, the proposed AHN-classifier ranks in second place with an accuracy of 98.76% such below to the deep learning based classifier. Then, mixture discriminant analysis, C5.0 decision trees, random forest and SVM with radial function are in the top of the list.

### 6.2. Case 2: User-Independent Performing Leave-One Subject-Out

**Figure 7:**
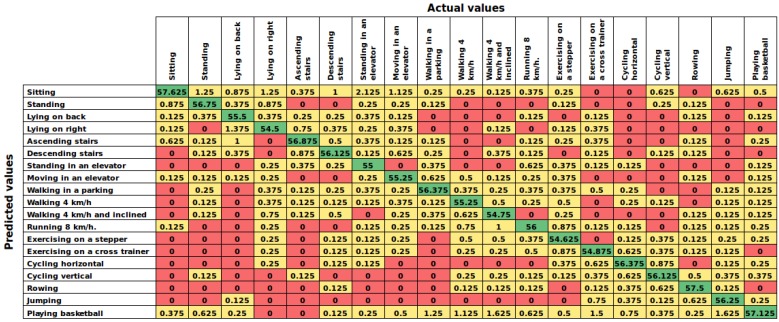
Results of case 2: Confusion matrix of the AHN-classifier in the leave-one subject-out performance for user-independent. Numbers represent the average of window counts in the eight models.

This experiment is based on the well-known leave-one subject-out technique [1], aiming to prove how well is the AHN-classifier to predict activities in new subjects. Table 7 shows the overall performance of the supervised models sorted in descending order by accuracy, and Table 8 shows the performance of each model. In addition, Figure 7 shows the average confusion matrix of the AHN-classifier. In this case, the AHN-classifier also ranks in second place with a mean accuracy of 93.23%±1.37% just below the deep learning based classifier. Additionally, penalized discriminant analysis, shrinkage discriminant analysis, mixture discriminant analysis and nearest shrunken centroids are also in the top of the list.

### 6.3. Case 3: User-Dependent Performing Cross-Validation within a Subject

**Figure 8:**
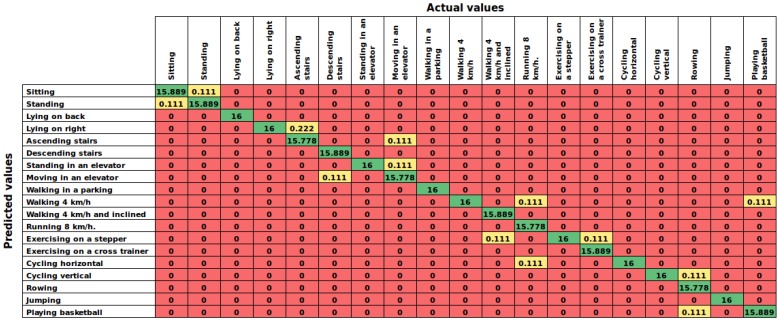
Results of case 3: Confusion matrix of the AHN-classifier in the cross-validation within a subject performance for user-dependent. Numbers represent the average of window counts in the eight models.

This experiment considers building eight different models from each of the subjects in order to measure the performance of the AHN-classifier in a user-specific approach. Table 9 summarizes the overall results of this experiment sorted in descending order by accuracy, and Table 10 shows the performance of each model. Additionally, Figure 8 reports the confusion matrix of the proposed AHN-classifier. The proposed AHN-classifier ranks at the first place with a mean accuracy of 99.49%±0.44%. Currently, deep learning, mixture discriminant analysis, shrinkage discriminant analysis and penalized discriminant analysis are also in the top of the list.

### 6.4. Discussion

As noted above, the proposed AHN-classifier outperformed in the three case experiments. In addition, we conducted a paired *t*-test analysis to find out if the differences between the accuracy of the AHN-classifier performance and the other supervised model performances are statistically significant. Table 11 summarizes the *p*-values of this test for cases 2 and 3 using a 95% confidence level. As shown, any *p*-value greater than 0.05 (bold values in Table 11) means that the null hypothesis about the equality of accuracy values between model performances is accepted, otherwise accuracy values between model performances are not statistically equal and the hypothesis is denied. In that sense, the AHN-classifier can be fairly compared with those model performances with a *p*-value less than 0.05, concluding that the AHN-classifier is significantly better than mixture discriminant analysis based classifier and those below the seventh position in Table 8 for case 2. In addition, the AHN-classifier is significantly better than those model performances below the third position in Table 10. To this end, it is shown that the AHN-classifier is significantly equivalent to deep learning in both cases 2 and 3. In fact, these experiments and their *t*-test analysis consider to validate that the AHN-classifier is suitable as a flexible approach for HAR systems based on: The ability to support new users (user-independent), and the ability to build models for a specific user. Furthermore, new and unknown activities need more tests before validating its flexibility. From now, we handle and filter them before the main human activity classification.

On one hand, the proposed AHN-classifier reached 98.76% (case 1) when dealing with a HAR system using all subjects for training and testing. In the user-independent performance (case 2), results computed 93.23%±1.37% of accuracy in cross-validation over subjects and leave-one subject-out experiments. Then, analyzing the confusion matrices of both experiments (Figure 6 and Figure 7), it can be seen that false predictions are very close to the diagonal (true positives). In the cross-validation experiment (case 1), we can observed that the activities predicted are very similar to the actual activities. For example, the AHN-classifier predicted lying on back when the actual activity was lying on right side. Likewise, it predicted walking at 4 km/h on flat when the actual activity was walking in a parking lot. In the leave-one subject-out experiment (case 2), the maximum average window counts value has to be 60, the number of window counts per activity, at each element in the chart of Figure 7. Then, it can be observed that true positive counts are very close to the maximum value and the others are close to zero. It means that the AHN-classifier is able to truly classify human activities with a very low misclassification.

On the other hand, in user-dependent approach, the proposed AHN-classifier reached 99.49%±0.44% of accuracy in cross-validation within a subject. In some cases such as *subject-4* and *subject-6*, the AHN-classifier predicts 100% of the activities carried out by the subject. This is a slight advantage over the other five top supervised models (see Table 9). The same behavior in the confusion matrix of this experiment (Figure 8) was found as in the other cases.

It is important to note that deep learning, i.e., DNN, based classifier is the only model that outperforms the AHN-classifier in the first two cases. Conversely, the AHN-classifier kept at the top of the benchmark experiments in comparison with deep learning which dropped to the second place in case 3.

In terms of the unknown-activity detection module using the AHN-classifier, the experimentation shows a suggested way in which the AHN-based classifier can discriminate known and unknown activities by themselves, using the centers of molecules. The latter actually reflects the parameter interpretability property of AHN, since the centers of molecules serve as features to find correspondence between training and new data. Thus, a proper training of a single AHN-classifier should deal with both human activity classification and detecting unknown activities at the same time. Particularly to this work, the centers of molecules were not employed in the main experimentation (cases 1, 2 and 3) since the sensor signals are clearly related to known activities in the dataset.

From Table 5, some computational issues can be identified in the AHN-classifier. Particularly, the training time of the AHN (1709.12 s) exceeded 17.2 times the maximum training time (99.215 s) performed by the other methods. This step is time-consuming mainly on the splitting procedure at each iteration of Algorithm 1 and the inner building function (1) used for running the LSE method. In the current work, this is not a problem. However, if real-time HAR systems are implemented, an improvement on this computational issue will be handled. For instance, another splitting procedure might be considered.

To this end, the AHN-classifier can achieve a flexible approach for user-dependent, user-independent, or both scenarios, in human activity recognition, as described in this work.

## 7. Conclusions and Future Work

In order to cope with real-world activity recognition challenges, a supervised machine learning technique must be flexible. In this paper, we considered flexibility of the approach regarding: The ability to support new users (user-independent). We were also concerned the ability to support variations of the same subject in a user-specific approach, and the ability to handle new or irrelevant activities.

In that sense, we presented a novel supervised machine learning method called artificial hydrocarbon networks as a flexible approach for human activity recognition. The AHN-classifier performance was compared with eighteen commonly used supervised techniques. We also designed an unknown-activity detection module that performs a rough classification to handle new and irrelevant activities. For our user-independent and user-dependent case scenarios, our results showed that AHN-classifier remained at the top of the other classifiers.

Our results demonstrated that artificial hydrocarbon networks classifier serves as a flexible approach when building a human activity recognition system with either user-dependent or user-independent approaches.

For future research, we must address flexibility regarding the ability to recognize new and complex activities. Also, the parameter interpretability of AHN will be deeply analyzed to determine the conditions and training procedures to perform human activity recognition and unknown-activity detection with a single AHN-based model. Further experimentation is also needed in order to prove flexibility when intra-subject variability occurs. Another challenge to be attended is to demonstrate that our AHN-classifier is well suited for real-time HAR systems, using other sensor configurations and improvements of computational issues at the training step of the method.

## Figures and Tables

**Table 1:** Description of the chemical terms used in artificial hydrocarbon networks.

Chemical Terminology	Symbols	Meaning
environment	*x*	(features) data inputs
behavior	*y*	(target) data outputs, solution of mixtures
atoms	$H_{i}$, *σ*	(parameters) basic structural units or properties
molecules	φ(x)	(functions) basic units of information
compounds	ψ(x)	(composite functions) complex units of information made of molecules
mixtures	S(x)	(linear combinations) combination of compounds
stoichiometric coefficient	$\alpha_{i}$	(weights) definite ratios in mixtures
intermolecular distances	$r_{j}$	(distances) length between two adjacent molecules
bounds	$L_{0},L_{j}$	(parameters) lower and upper delimiters, in the inputs, of molecules
energy	$E_{0},E_{j}$	(loss function) value of the error between real and estimated values

**Table 2:** Features extracted in time domain.

Features	References
mean	[4,29,30,31,32,33,34]
standard deviation	[30,31,34]
root mean square	[29]
maximal amplitude	[31,33]
minimal amplitude	[31,33]
median	[32,34]
number of zero-crossing	[29,31]
skewness	[33]
kurtosis	[4,33]
first quartile	[32,34]
third quartile	[32,34]
autocorrelation	[31,33]

**Table 3:** Features extracted in frequency domain.

Features	References
mean frequency	[29,31]
median frequency	[29]
entropy	[30,32]
energy	[4,30,32]
principal frequency	[32,33,34]
spectral centroid	[31,32]

**Table 4:** Accuracy of the unknown-activity detection module.

Activity	Target	Accuracy of AHN	Accuracy of *k*-Means
sitting	*known*	0.9583	0.9653
lying on back	*known*	0.9166	0.8958
ascending stairs	*known*	0.9306	0.8472
walking in a parking lot	*known*	0.6042	0.9444
exercising on a stepper	*known*	0.9583	0.9653
cycling on an exercise bike	*unknown*	0.1666	0.9583
jumping	*unknown*	0.7917	1.0000
walking on a treadmill	*unknown*	0.0208	0.1528
lying on right side	*unknown*	1.0000	1.0000

**Table 5:** Configuration parameters for building suitable activity models using the caret package in R. Other parameters of the method marked with (*) are: Activation_function = hyperbolic tangent, hidden_layers = (200, 250, 200), balance_classes = true.

No	Method	Configurations	Parameters	Values	Training Time (s)	Testing Time (ms)
1	AdaBoost	27	(mfinal, maxdepth, coeflearn)	(150, 3, 3)	22.960	1.213
**2**	**Artificial Hydrocarbon Networks**	**1**	**(n_molecules, learning_rate, tolerance)**	**(19, 0.5, 0.1)**	**1709.120**	**0.028**
3	C4.5-Decision Trees	1	(C)	(0.25)	3.426	0.069
4	C5.0-Decision Trees	12	(trials, model, winnow)	(20, 1, TRUE)	10.509	0.545
5	Deep Learning *	1	(rate annealing, epochs, rate)	(0.001, 300, 0.01)	21.580	0.970
6	k-Nearest Neighbors	3	(kmax, distance, kernel)	(5, 2, 1)	5.777	0.804
7	Mixture Discriminant Analysis	3	(subclasses)	(4)	5.839	0.197
8	Model Averaged Artificial Neural Networks	9	(size, decay, bag)	(5, 0.1, FALSE)	12.114	0.040
9	Multivariate Adaptive Regression Splines	1	(degree)	(1)	99.215	0.172
10	Naive Bayes	2	(fL, usekernel)	(0, TRUE)	32.065	92.953
11	Nearest Shrunken Centroids	3	(threshold)	(3.38)	0.069	0.022
12	Artificial Neural Networks	9	(size, decay)	(5, 0.1)	5.905	0.022
13	Penalized Discriminant Analysis	3	(lambda)	(1)	0.364	0.022
14	Random Forest	3	(mtry, ntrees)	(2, 100)	29.464	0.077
15	Rule-Based Classifier	1	(threshold, pruned)	(0.25, 1)	7.213	0.077
16	Shrinkage Discriminant Analysis	3	(diagonal, lambda)	(FALSE, 0)	0.299	0.018
17	Single Rule Classification	1	(-)	(-)	2.980	0.062
18	Stochastic Gradient Boosting	9	(n.trees, interaction.depth, shrinkage)	(150, 3, 0.1)	18.277	0.164
19	SVM with Radial Basis Function Kernel	3	(C)	(1)	25.479	3.187

**Table 6:** Results of case 1: Performance for all subjects using cross-validation.

No	Method	Accuracy	Sensitivity	Specificity	Precision	$F_{1}$-Score
1	Deep Learning	99.27	99.27	99.96	99.28	99.62
**2**	**Artificial Hydrocarbon Networks**	**98.76**	**98.76**	**99.93**	**98.78**	**99.35**
3	Mixture Discriminant Analysis	98.36	98.36	99.91	98.43	99.16
4	C5.0-Decision Trees	98.28	98.28	99.90	98.28	99.08
5	Random Forest	98.25	98.25	99.90	98.27	99.08
6	SVM with Radial Basis Function Kernel	98.10	98.10	99.89	98.17	99.03
7	Stochastic Gradient Boosting	97.99	97.99	99.89	98.03	98.95
8	Artificial Neural Networks	97.88	97.88	99.88	97.87	98.87
9	Multivariate Adaptive Regression Splines	97.48	97.48	99.86	97.43	98.63
10	Penalized Discriminant Analysis	97.00	97.00	99.83	97.07	98.43
11	Shrinkage Discriminant Analysis	97.00	97.00	99.83	97.07	98.43
12	Rule-Based Classifier	96.27	96.27	99.79	96.29	98.01
13	k-Nearest Neighbors	95.76	95.76	99.76	95.67	97.68
14	Naive Bayes	95.58	95.58	99.75	95.86	97.77
15	AdaBoost	95.50	95.50	99.75	95.78	97.73
16	C4.5-Decision Trees	95.25	95.25	99.74	95.25	97.44
17	Nearest Shrunken Centroids	93.31	93.31	99.63	93.70	96.57
18	Model Averaged Artificial Neural Networks	91.05	91.05	99.50	92.70	95.98
19	Single Rule Classification	37.87	37.87	96.55	38.05	54.59
	**Average**	**93.32**	**93.32**	**99.63**	**93.48**	**95.82**

**Table 7:** Results of case 2: Leave-one subject-out overall performance for the user-independent approach. Values with (*) were obtained using only available metrics when they can be performed over results.

No	Method	Accuracy	Sensitivity	Specificity	Precision	$F_{1}$-Score
1	Deep Learning	94.05	94.05	99.67	96.04	97.82
**2**	**Articial Hydrocarbon Networks**	**93.23**	**93.23**	**99.62**	**93.59**	**96.51**
3	Penalized Discriminant Analysis	92.64	92.64	99.59	94.28 *	96.87 *
4	Shrinkage Discriminant Analysis	92.63	92.63	99.59	94.28 *	96.87 *
5	Mixture Discriminant Analysis	90.80	90.80	99.49	92.26 *	95.73 *
6	Nearest Shrunken Centroids	90.41	90.41	99.47	91.39 *	95.23 *
7	C5.0-Decision Trees	87.57	87.57	99.31	89.37 *	94.07 *
8	Random Forest	87.35	87.35	99.30	90.00 *	94.39 *
9	Stochastic Gradient Boosting	87.11	87.11	99.28	90.64 *	94.75 *
10	AdaBoost	86.80	86.80	99.27	88.40 *	93.43 *
11	Multivariate Adaptive Regression Splines	85.15	85.15	99.18	86.69 *	92.46 *
12	SVM with Radial Basis Function Kernel	81.33	81.33	98.96	88.44 *	93.36 *
13	Rule-Based Classifier	81.23	81.23	98.96	84.13 *	90.93 *
14	C4.5-Decision Trees	80.07	80.07	98.89	83.93 *	90.79 *
15	Naive Bayes	79.06	79.06	98.84	70.01 *	74.29 *
16	Model Averaged Artificial Neural Networks	75.04	75.04	98.61	77.45 *	86.82 *
17	k-Nearest Neighbors	74.91	74.91	98.61	82.18 *	89.65 *
18	Artificial Neural Networks	61.38	61.38	97.85	76.32 *	86.06 *
19	Single Rule Classification	29.92	29.92	96.11	30.79	46.51
	**Average**	**80.92**	**80.92**	**98.94**	84.22 *	89.82 *

**Table 8:** Results of case 2: Leave-one subject-out performance for the user-independent approach for each of the models created.

No	Method	Avg & Std	Sub 1	Sub 2	Sub 3	Sub 4	Sub 5	Sub 6	Sub 7	Sub 8
1	Deep Learning	94.05 ± 1.61	97.37	95.35	92.54	92.54	93.60	93.95	93.42	93.60
**2**	**Articial Hydrocarbon Networks**	**93.23 ± 1.37**	**94.56**	**95.61**	**92.54**	**92.63**	**92.46**	**92.63**	**94.04**	**91.40**
3	Penalized Discriminant Analysis	92.64 ± 0.88	93.42	92.46	92.46	91.93	94.04	91.14	92.81	92.89
4	Shrinkage Discriminant Analysis	92.63 ± 0.88	93.42	92.46	92.46	91.93	94.04	91.14	92.72	92.89
5	Mixture Discriminant Analysis	90.8 ± 1.82	90.09	89.39	90.26	89.39	93.95	89.39	93.33	90.61
6	Nearest Shrunken Centroids	90.41 ± 3.38	85.26	93.60	93.95	91.84	93.16	90.88	88.16	86.40
7	C5.0-Decision Trees	87.57 ± 4.35	82.81	86.75	90.44	80.79	87.98	89.65	94.65	87.46
8	Random Forest	87.35 ± 3.94	83.25	84.91	86.84	90.70	91.93	81.49	87.98	91.67
9	Stochastic Gradient Boosting	87.11 ± 5.09	84.30	83.33	91.75	81.93	95.09	83.68	92.37	84.39
10	AdaBoost	86.8 ± 5.1	81.14	86.58	93.95	78.77	89.82	85.26	91.58	87.28
11	Multivariate Adaptive Regression Splines	85.15 ± 4.72	82.63	85.53	87.72	90.35	80.70	76.93	90.00	87.37
12	SVM with Radial Basis Function Kernel	81.33 ± 3.1	76.49	80.61	80.70	86.14	82.98	78.60	80.70	84.39
13	Rule-Based Classifier	81.23 ± 6.17	75.09	82.46	85.18	69.21	81.05	86.75	87.02	83.07
14	C4.5-Decision Trees	80.07 ± 5.17	77.89	77.81	77.19	73.68	84.82	86.67	86.67	75.79
15	Naive Bayes	79.06 ± 4.41	73.77	76.84	84.30	81.23	82.46	72.11	79.39	82.37
16	Model Averaged Artificial Neural Networks	76.82 ± 10.46	73.68	64.04	96.32	78.33	73.60	71.67	87.63	69.30
17	k-Nearest Neighbors	74.91 ± 6.13	74.12	75.79	72.54	77.11	85.00	63.33	78.33	73.07
18	Artificial Neural Networks	73.65 ± 8.97	75.53	65.18	85.70	81.32	78.95	61.14	64.30	77.11
19	Single Rule Classification	29.92 ± 4.14	30.09	24.21	29.47	28.16	32.89	37.54	25.96	31.05
	**Average**	**81.7 ± 4.44**	**79.31**	**79.86**	**84.65**	**80.86**	**84.16**	**79.44**	**83.76**	**81.58**

**Table 9:** Results of case 3: Cross-validation within a subject overall performance for the user-dependent approach. Values with (*) were obtained using only available metrics when they can be performed over results.

No	Method	Accuracy	Sensitivity	Specificity	Precision	$F_{1}$-Score
**1**	**Articial Hydrocarbon Networks**	**99.49**	**99.49**	**99.97**	**99.51**	**99.74**
2	Deep Learning	99.27	99.27	99.96	99.35	99.66
3	Mixture Discriminant Analysis	99.20	99.20	99.96	99.26	99.61
4	Shrinkage Discriminant Analysis	99.05	99.05	99.95	99.12	99.53
5	Penalized Discriminant Analysis	99.01	99.01	99.95	99.08	99.51
6	Model Averaged Artificial Neural Networks	98.79	98.79	99.93	98.84	99.38
7	Random Forest	98.72	98.72	99.93	98.79	99.36
8	Multivariate Adaptive Regression Splines	98.43	98.43	99.91	98.59	99.25
9	C5.0-Decision Trees	97.99	97.99	99.89	98.07	98.97
10	SVM with Radial Basis Function Kernel	97.92	97.92	99.88	98.06	98.96
11	Nearest Shrunken Centroids	97.62	97.62	99.87	97.91	98.88
12	Stochastic Gradient Boosting	97.48	97.48	99.86	97.64	98.73
13	AdaBoost	97.44	97.44	99.86	97.96	98.90
14	Naive Bayes	97.26	97.26	99.85	97.70	98.76
15	C4.5-Decision Trees	96.42	96.42	99.80	96.61	98.18
16	Rule-Based Classifier	95.94	95.94	99.77	96.09	97.89
17	k-Nearest Neighbors	95.61	95.61	99.76	95.67	97.67
18	Artificial Neural Networks	92.58	92.58	99.59	48.12 *	48.98 *
19	Single Rule Classification	61.84	61.84	97.88	54.11	66.27
	**Average**	**95.79**	**95.79**	**99.77**	**95.69**	**97.18**

**Table 10:** Results of case 3: Cross-validation within a subject performance for the user-dependent approach for each of the models created.

No	Method	Avg & Std	Sub 1	Sub 2	Sub 3	Sub 4	Sub 5	Sub 6	Sub 7	Sub 8
**1**	**Articial Hydrocarbon Networks**	**99.49 ± 0.44**	**99.12**	**99.12**	**99.71**	**100.00**	**98.83**	**100.00**	**99.42**	**99.71**
2	Deep Learning	99.27 ± 0.16	99.12	99.42	99.12	99.42	99.42	99.12	99.12	99.42
3	Mixture Discriminant Analysis	99.2 ± 0.46	98.54	98.83	99.42	99.42	98.83	99.42	99.12	100.00
4	Shrinkage Discriminant Analysis	99.05 ± 0.3	98.54	99.12	99.42	99.42	98.83	99.12	98.83	99.12
5	Penalized Discriminant Analysis	99.01 ± 0.38	98.25	99.12	99.42	99.42	98.83	99.12	98.83	99.12
6	Model Averaged Artificial Neural Networks	98.79 ± 0.36	98.25	98.54	98.54	99.42	98.83	98.83	99.12	98.83
7	Random Forest	98.72 ± 0.62	98.25	97.95	98.54	99.42	98.25	98.54	99.12	99.71
8	Multivariate Adaptive Regression Splines	98.43 ± 1.34	95.61	98.54	99.71	99.12	97.37	99.12	99.42	98.54
9	C5.0-Decision Trees	97.99 ± 0.57	96.78	98.25	97.66	98.54	98.25	97.95	97.95	98.54
10	SVM with Radial Basis Function Kernel	97.92 ± 0.74	97.66	96.78	97.37	98.83	97.66	98.54	97.66	98.83
11	Nearest Shrunken Centroids	97.62 ± 1.1	97.66	97.37	96.20	98.83	95.91	97.95	98.25	98.83
12	Stochastic Gradient Boosting	97.48 ± 1.12	95.61	97.08	97.37	99.12	98.54	96.49	97.66	97.95
13	AdaBoost	97.44 ± 1.32	95.91	97.95	98.25	98.83	97.66	95.03	97.37	98.54
14	Naive Bayes	97.26 ± 1.06	96.78	96.20	97.95	99.42	96.20	96.78	97.37	97.37
15	C4.5-Decision Trees	96.42 ± 0.84	95.32	96.20	97.08	97.08	97.08	95.32	97.37	95.91
16	Rule-Based Classifier	95.94 ± 1.26	95.03	95.32	95.32	95.91	96.49	94.15	97.95	97.37
17	k-Nearest Neighbors	95.61 ± 1.04	96.78	93.86	96.49	95.32	95.03	94.74	96.49	96.20
18	Neural Network	92.58 ± 4.16	89.18	94.15	96.49	98.25	85.09	93.86	91.23	92.40
19	Single Rule Classification	61.84 ± 4.1	63.45	65.20	63.74	62.87	67.54	57.89	58.48	55.56
	**Average**	**95.79 ± 1.12**	**95.04**	**95.74**	**96.2**	**96.77**	**95.51**	**95.37**	**95.83**	**95.89**

**Table 11:** Results of the *t*-test analysis reporting the *p*-values or cases 2 and 3. Bold values represent *p*-values greater than 0.05 (95% confidence level).

Method	*p*-Value in Case 2	*p*-Value in Case 3
AdaBoost	0.013	0.003
C4.5-Decision Trees	0.000	0.000
C5.0	0.010	0.000
Deep Learning	**0.109**	**0.244**
k-Nearest Neighbors	0.000	0.000
Mixture Discriminant Analysis	0.025	0.000
Model Averaged Artificial Neural Networks	0.004	0.004
Multivariate Adaptive Regression Splines	0.002	0.002
Naive Bayes	0.000	0.006
Nearest Shrunken Centroids	**0.063**	0.001
Artificial Neural Networks	0.001	0.002
Penalized Discriminant Analysis	**0.324**	0.000
Random Forest	0.011	0.033
Rule-Based Classifier	0.001	0.005
Shrinkage Discriminant Analysis	**0.317**	0.000
Single Rule Classification	0.000	0.000
Stochastic Gradient Boosting	0.016	0.001
SVM with Radial Basis Function Kernel	0.000	0.031

## Appendix A. Numerical Example of Artificial Hydrocarbon Networks

This section shows the training and testing steps of the artificial hydrocarbon networks algorithm (AHN-algorithm) for classification purposes. In order to do that, a simple numerical example was selected.

### Appendix A.1. Training Step

Consider the 20-sample dataset provided in Table A1. Each sample has three features associated, namely $x_{1},x_{2},x_{3}$, and its proper label *y*. Then, training a classifier model using the AHN-algorithm requires the following steps: (i) define the training set; (ii) set the configuration parameters; (iii) run the AHN-algorithm; and (iv) obtain the AHN-classifier model.

**Table A1:** Data set used in the numerical example.

No Sample	$x_{1}$	$x_{2}$	$x_{3}$	*y*	No Sample	$x_{1}$	$x_{2}$	$x_{3}$	*y*
1	4.3	3.6	5.1	1	11	6.9	4.9	3.8	2
2	4.2	2.4	6.8	1	12	7.1	2.5	3.7	2
3	3.9	6.2	6.8	1	13	7.5	4.7	−4.3	2
4	3.8	3.6	6.5	1	14	7.8	5.1	3.4	2
5	3.3	5.6	6.3	1	15	6.5	6.5	0.7	2
6	3.7	4.5	6.6	1	16	6.8	15.7	−3.0	3
7	3.6	5.9	6.4	1	17	7.5	17.2	−3.2	3
8	4.4	3.6	5.4	1	18	7.2	16.9	−2.3	3
9	4.5	2.0	5.5	1	19	6.9	16.3	−2.2	3
10	4.9	5.2	5.3	1	20	7.0	17.1	0.4	3

#### Definition of the Training Set

For this particular example, the training set is defined to be 50% of the original dataset and the remaining 50% will be considered for testing. For example, the collection of samples in these training and testing sets were set as summarized in Table A2, using random selection.

**Table A2:** Training and testing sets for the numerical example.

Set	Samples
*training*	{1,6,9,10,13,15,16,18,19,20}
*testing*	{2,3,4,5,7,8,11,12,14,17}

#### Setting the Configuration Parameters

As written in Algorithm 1, three configuration parameters are required: The number of molecules in the hydrocarbon compound *n*, the learning rate *η* and the tolerance value *ϵ*. For this numerical example, the following parameters were selected: n=3, η=0.1 and ϵ=0.01.

#### Running the AHN-Algorithm

Then, the AHN-algorithm is computed. The next description is based on Algorithm 1. First, an empty structure is initialized AHN={} in which the final result will be stored. Then, a saturated and linear hydrocarbon compound *C* is created. Since the number of molecules n=3, then the shape of the compound is like $CH_{3}-CH_{2}-CH_{3}$. This configuration of the compound shows that the first molecule is composed of three hydrogen values, the next one with two hydrogen values and the last molecule is composed of three hydrogen values. Following the algorithm, a set of intermolecular distances $r_{j}$ for j=1,2,3 has to be randomly generated, as shown in Table A3 when i=0. Notice that each distance is a vector in the feature space, such that $r_{j}={\left\{r_{1},r_{2},r_{3}\right\}}_{j}$.

After that, a while-loop starts until the stop criterion holds, i.e., ∥y−ψ∥≤ϵ. Inside the loop, the set of bounds $L_{j}$ is calculated using $L_{j}=L_{j-1}+r_{j}$ considering that $L_{0}$ is the minimum values of each feature $L_{min}$, all $L_{j}$ is less or equals to the maximum values of each feature $L_{max}$, and $L_{n}=L_{max}$. For instance, $L_{min}=\left(3.3,2.0,-4.3\right)$ and $L_{max}=\left(7.8,17.2,6.8\right)$. Then, the set of $L_{j}$ at the first iteration i=0 is shown in Table A3.

**Table A3:** Intermolecular distances and bounds for the numerical example at iterations i=0 and i=1.

*j*	$r_{j}\left(i=0\right)$	$L_{j}\left(i=0\right)$	$r_{j}\left(i=1\right)$
0	−	(3.3,2.0,−4.3)	−
1	(0.7,3.2,2.5)	(4.0,5.2,−1.8)	(0.7,3.2,2.5)
2	(1.3,8.2,4.2)	(5.3,13.4,2.4)	(1.31,8.21,4.21)
3	(2.2,5.7,7.8)	(7.8,17.2,6.8)	(2.19,5.69,7.79)

Currently, this set of bounds is employed for partitioning the training set into *n* subsets, $\Sigma_{j}$ for j=1,2,3, each one containing a subset of samples clustered by the input domain such as (A1) [23]. For this example, the partition of the training set at iteration i=0 is summarized in Table A4.

(A1) $$\Sigma_{t}=\left\{\left(x^{\left(q\right)},y^{\left(q\right)}\right)|t=\arg\min_{j}\left(∥x^{\left(q\right)}-\frac{L_{j-1}+L_{j}}{2}∥\right)\;\mathrm{and}\;j=1,\cdots ,n\right\}$$

**Table A4:** Obtained subsets when partitioning the training set at iteration i=0.

Set	Samples
$\Sigma_{1}$	{9,12,13}
$\Sigma_{2}$	{1,2,3,4,5,6,7,8,10,11,14,15,16}
$\Sigma_{3}$	{17,18,19,20}

For each CH-molecule *j*, it is computed all hydrogen $H_{i}$ and carbon $\sigma_{r}$ values of function for a multivariate case, as shown in (A2), using the least squares estimates (LSE) method with subset $\Sigma_{j}$ as expressed in (A3). Additionally, each error estimate $E_{j}$ should be stored. For this example, the obtained error values after computing the LSE method are: E={0.0,0.0,0.0084,0.0}. Notice that $E_{0}=0.0$.

(A2) $$\varphi_{j}\left(x\right)=\sum_{r=1}^{n}\sigma_{r}\prod_{i=1}^{k\leq 4}\left(x_{r}-H_{ir}\right)$$

(A3) $$E_{j}=\frac{1}{2}\sum {∥y-\varphi_{j}\left(x\right)∥}^{2},\;\mathrm{for}\;\mathrm{all}\;\left(x,y\right)\in \Sigma_{j}$$

The next step in the algorithm is to build the compound behavior ψ(x). For this example, the resultant molecular functions $\varphi_{j}\left(x\right)$ are those shown in (A4); where $x\in \Sigma_{j}$ denotes that an input *x* classifies in $\Sigma_{j}$ using the condition (A1).

(A4) $$\psi \left(x\right)=\left\{\begin{array}{ll} \varphi_{1}\left(x\right)=0.0051x_{1}^{3}+0.0010x_{2}^{3}+0.0032x_{3}^{3} & x\in \Sigma_{1}\\ \varphi_{2}\left(x\right)=0.0438\left(x_{1}-5.5205\right)x_{1}-0.0013\left(x_{2}-18\right)x_{2}+\;\;0.0186\left(x_{3}-5.64-7.96i\right)\left(x_{3}-5.64+7.96i\right) & x\in \Sigma_{2}\\ \varphi_{3}\left(x\right)=-0.0004x_{1}^{3}-0.0013\left(x_{2}-25\right)x_{2}^{2}-0.0009x_{3}^{3} & x\in \Sigma_{3} \end{array}\right.$$

Then, the intermolecular distances have to be updated using (4) and (5). In this case, $r_{j}$ is updated using the learning rate η=0.1 such that $r_{j}=r_{j}-0.1\left(E_{j-1}-E_{j}\right)$. Table A3 summarizes this step when i=1. After that, in order to determine the stop criterion, the difference ∥y−ψ∥ is estimated by computing the overall error $E_{global}=\sum_{j}\left(E_{j}\right)$. Lastly, the criterion is verified: If $E_{global}\leq \epsilon$, then the while-loop finishes. In this case, $E_{global}=0.0084<0.01$ stopping the algorithm.

Once the while-loop ends, the AHN structure is already completed. So, AHN=〈C,ψ(x)〉; where, *C* is of the form as $CH_{3}-CH_{2}-CH_{3}$ and ψ(x) is equals to (A4).

#### Obtaining the AHN-Classifier Model

To this end, the AHN-classifier model is defined by the AHN structure obtained from the AHN-algorithm. Also, a rounding process is computed since artificial hydrocarbon networks is primary a regressor. For this example, the obtained AHN-classifier $y_{AHN}$ is the one expressed in (A5); where, ψ(x) is the compound behavior of (A4). A good practice to ensure this thresholding process is to modify (A3) with (A6). In that sense, the training process will be computed in terms of the output rounding estimation.

(A5) $$y_{AHN}\left(x\right)=\mathrm{round}\left(\psi \left(x\right)\right)$$

(A6) $$E_{j}=\frac{1}{2}\sum {∥y-\mathrm{round}\left(\varphi_{j}\left(x\right)\right)∥}^{2},\;\mathrm{for}\;\mathrm{all}\;\left(x,y\right)\in \Sigma_{j}$$

### Appendix A.2. Testing Step

This step considers to validate the AHN-classifier $y_{AHN}$ already developed, using the testing set (Table A2). For instance, the first sample in the testing set is x=(4.2,2.4,6.8) with y=1. Then, the input of the AHN-classifier is *x* and the expected value $y_{AHN}\left(x\right)$ should be similar to y=1. In this case, $y_{AHN}\left(x\right)=\mathrm{round}\left(1.0091\right)=1$. Table A5 summarizes the evaluation of all the testing set and it is compared with the original values. To this end, Table A5 shows that the testing set is estimated accordingly with the AHN-classifier. For an extended description of training and testing artificial hydrocarbon networks, see [23,25].

**Table A5:** Comparison between estimated $y_{AHN}$ and target *y* values for the numerical example.

No Sample	*y*	$y_{AHN}$
2	1	1
3	1	1
4	1	1
5	1	1
7	1	1
8	1	1
11	2	2
12	2	2
14	2	2
17	3	3

## References

[1] Lara O.D., Labrador M.A. (2013). A survey on human activity recognition using wearable sensors. IEEE Commun. Surv. Tutor..

[2] Kim E., Helal S., Cook D. (2010). Human activity recognition and pattern discovery. IEEE Pervasive Comput..

[3] Berchtold M., Budde M., Schmidtke H.R., Beigl M. (2010). An extensible modular recognition concept that makes activity recognition practical. KI 2010: Advances in Artificial Intelligence.

[4] Bulling A., Blanke U., Schiele B. (2014). A tutorial on human activity recognition using body-worn inertial sensors. ACM Comput. Surv..

[5] Reiss A. (2014). Personalized Mobile Physical Activity Monitoring for Everyday Life. Ph.D. Thesis.

[6] Ponce H., Martinez-Villaseñor L., Miralles-Pechuan L. (2015). Comparative analysis of artificial hydrocarbon networks and data-driven approaches for human activity recognition. Lecture Notes in Computer Science.

[7] Ponce H., Martinez-Villaseñor L., Miralles-Pechuan L. (2016). A novel wearable sensor-based human activity recognition approach using artificial hydrocarbon networks. Sensors.

[8] Attal F., Mohammed S., Dedabrishvili M., Chamroukhi F., Oukhellou L., Amirat Y. (2015). Physical human activity recognition using wearable sensors. Sensors.

[9] Lin W., Sun M.T., Poovandran R., Zhang Z. Human activity recognition for video surveillance. Proceedings of the IEEE International Symposium on Circuits and Systems.

[10] Zhu C., Sheng W. Multi-sensor fusion for human daily activity recognition in robot-assisted living. Proceedings of the 4th ACM/IEEE International Conference on Human Robot Interaction.

[11] Minnen D., Westeyn T., Ashbrook D., Presti P., Starner T. (2007). Recognizing soldier activities in the field. Proceedings of the 4th International Workshop on Wearable and Implantable Body Sensor Networks (BSN 2007).

[12] Capela N., Lemaire E., Baddour N., Rudolf M., Goljar N., Burger H. (2016). Evaluation of a smartphone human activity recognition application with able-bodied and stroke participants. J. Neuroeng. Rehabil..

[13] Tapia E.M., Intille S.S., Haskell W., Larson K., Wright J., King A., Friedman R. Real-time recognition of physical activities and their intensities using wireless accelerometers and a heart rate monitor. Proceedings of the 2007 11th IEEE International Symposium on Wearable Computers.

[14] Parkka J., Cluitmans L., Ermes M. (2010). Personalization algorithm for real-time activity recognition using PDA, wireless motion bands, and binary decision tree. IEEE Trans. Inf. Technol. Biomed..

[15] Bleser G., Steffen D., Reiss A., Weber M., Hendeby G., Fradet L. (2015). Personalized physical activity monitoring using wearable sensors. Smart Health.

[16] Cook D., Feuz K., Krishnan N. (2013). Transfer learning for activity recognition: A survey. Knowl. Inf. Syst..

[17] Byrnes J. (2001). Cognitive Development and Learning in Instructional Contexts.

[18] Cook D.J., Krishnan N.C. (2015). Activity Learning: Discovering, Recognizing, and Predicting Human Behavior from Sensor Data.

[19] Roggen D., Forster K., Calatroni A., Troster G. (2013). The adARC pattern analysis architecture for adaptive human activity recognition systems. J. Ambient Intell. Humaniz. Comput..

[20] Guo J., Zhou X., Sun Y., Ping G., Zhao G., Li Z. (2016). Smarthphone-based patients’ activity recognition by using a self-learning scheme for medical monitoring. J. Med. Syst..

[21] Li Z., Xie X., Zhou X., Guo J., Bie R. A generic framework for human motion recognition based on smartphones. Proceedings of the 2015 International Conference on Identification, Information, and Knowledge in the Internet of Things (IIKI).

[22] Preece S.J., Goulermas J.Y., Kenney L.P., Howard D., Meijer K., Crompton R. (2009). Activity identification using body-mounted sensors-a review of classification techniques. Physiol. Meas..

[23] Ponce H., Ponce P., Molina A. (2014). Artificial Organic Networks: Artificial Intelligence Based on Carbon Networks.

[24] Ponce H., Ponce P., Molina A. (2013). Artificial hydrocarbon networks fuzzy inference system. Math. Probl. Eng..

[25] Ponce H., Ponce P., Molina A. (2015). The development of an artificial organic networks toolkit for LabVIEW. J. Comput. Chem..

[26] Ponce H., Ponce P., Molina A. (2015). A novel robust liquid level controller for coupled-tanks systems using artificial hydrocarbon networks. Expert Syst. Appl..

[27] Ponce H., Ponce P., Molina A. (2014). Adaptive noise filtering based on artificial hydrocarbon networks: An application to audio signals. Expert Syst. Appl..

[28] Barshan B., Yüksek M.C. (2014). Recognizing daily and sports activities in two open source machine learning environments using body-worn sensor units. Comput. J..

[29] Phinyomark A., Nuidod A., Phukpattaranont P., Limsakul C. (2012). Feature extraction and reduction of wavelet transform coefficients for EMG pattern classification. Elektron. Elektrotech..

[30] Avci A., Bosch S., Marin-Perianu M., Marin-Perianu R., Havinga P. Activity recognition using inertial sensing for healthcare, wellbeing and sports applications: A survey. Proceedings of the 23rd International Conference on Architecture of Computing Systems (ARCS).

[31] Dargie W. Analysis of time and frequency domain features of accelerometer measurements. Proceedings of the 18th Internatonal Conference on Computer Communications and Networks.

[32] Rasekh A., Chen C.A., Lu Y. (2014). Human activity recognition using smartphone. Comput. Res. Repos..

[33] Atallah L., Lo B., King R., Yang G.Z. Sensor placement for activity detection using wearable accelerometers. Proceedings of the IEEE 2010 International Conference on Body Sensor Networks.

[34] Preece S.J., Goulermas J.Y., Kenney L.P., Howard D. (2009). A comparison of feature extraction methods for the classification of dynamic activities from accelerometer data. IEEE Trans. Biomed. Eng..

[35] Jolliffe I. (2002). Principal Component Analysis.

[36] Kaiser H.F. (1960). The application of electronic computers to factor analysis. Educ. Psychol. Meas..

[37] Roggen D., Calatroni A., Rossi M., Holleczek T., Förster K., Tröster G., Lukowicz P., Bannach D., Pirkl G., Ferscha A. Collecting complex activity datasets in highly rich networked sensor environments. Proceedings of the IEEE Seventh International Conference on Networked Sensing Systems (INSS).

[38] Dohnálek P., Gajdoš P., Moravec P., Peterek T., SnáŠel V. (2013). Application and comparison of modified classifiers for human activity recognition. Prz. Elektrotech..

[39] Sokolova M., Lapalme G. (2009). A systematic analysis of performance measures for classification tasks. Inf. Process. Manag..

